# Detection and diagnosis of automated breast ultrasound in patients with BI-RADS category 4 microcalcifications: a retrospective study

**DOI:** 10.1186/s12880-024-01287-4

**Published:** 2024-05-28

**Authors:** Li-Fang Yu, Chao-Chao Dai, Luo-Xi Zhu, Xiao-Jing Xu, Hong-Ju Yan, Chen-Xiang Jiang, Ling-Yun Bao

**Affiliations:** Department of Ultrasound, Hangzhou First People’s Hospital, No.261 Huansha Road, Hangzhou, 310006 Zhejiang Province China

**Keywords:** Automated breast ultrasound, Mammopgraphy, Microcalcification, Breast imaging reporting and Data System

## Abstract

**Background:**

Automated Breast Ultrasound (AB US) has shown good application value and prospects in breast disease screening and diagnosis. The aim of the study was to explore the ability of AB US to detect and diagnose mammographically Breast Imaging Reporting and Data System (BI-RADS) category 4 microcalcifications.

**Methods:**

575 pathologically confirmed mammographically BI-RADS category 4 microcalcifications from January 2017 to June 2021 were included. All patients also completed AB US examinations. Based on the final pathological results, analyzed and summarized the AB US image features, and compared the evaluation results with mammography, to explore the detection and diagnostic ability of AB US for these suspicious microcalcifications.

**Results:**

250 were finally confirmed as malignant and 325 were benign. Mammographic findings including microcalcifications morphology (61/80 with amorphous, coarse heterogeneous and fine pleomorphic, 13/14 with fine-linear or branching), calcification distribution (189/346 with grouped, 40/67 with linear and segmental), associated features (70/96 with asymmetric shadow), higher BI-RADS category with 4B (88/120) and 4 C (73/38) showed higher incidence in malignant lesions, and were the independent factors associated with malignant microcalcifications. 477 (477/575, 83.0%) microcalcifications were detected by AB US, including 223 malignant and 254 benign, with a significantly higher detection rate for malignant lesions (*x*^2^ = 12.20, *P* < 0.001). Logistic regression analysis showed microcalcifications with architectural distortion (odds ratio [OR] = 0.30, *P* = 0.014), with amorphous, coarse heterogeneous and fine pleomorphic morphology (OR = 3.15, *P* = 0.037), grouped (OR = 1.90, *P* = 0.017), liner and segmental distribution (OR = 8.93, *P* = 0.004) were the independent factors which could affect the detectability of AB US for microcalcifications. In AB US, malignant calcification was more frequent in a mass (104/154) or intraductal (20/32), and with ductal changes (30/41) or architectural distortion (58/68), especially with the both (12/12). BI-RADS category results also showed that AB US had higher sensitivity to malignant calcification than mammography (64.8% vs. 46.8%).

**Conclusions:**

AB US has good detectability for mammographically BI-RADS category 4 microcalcifications, especially for malignant lesions. Malignant calcification is more common in a mass and intraductal in AB US, and tend to associated with architectural distortion or duct changes. Also, AB US has higher sensitivity than mammography to malignant microcalcification, which is expected to become an effective supplementary examination method for breast microcalcifications, especially in dense breasts.

## Introduction

Breast microcalcification is one of the important signs of breast cancer. Mammography is the gold standard for detecting breast microcalcifications, it has been widely accepted in western countries for breast cancer screening strategies, which leads to earlier detection and reduce mortality from breast cancer in asymptomatic women [1–4]. The American College of Radiology (ACR) Breast Imaging Reporting and Data System (BI-RADS) has formulated established diagnostic criteria and recommended protocols based on the morphologic and distribution characteristics of calcifications on mammography, to assist in assessing the risk of malignancy and provide additional management recommendations [5]. Of which, BI-RADS category 4 is a suspicious assessment, and its malignant risk varies greatly with a range of 3-94%. That is to say there is a certain proportion of benign lesions, ultimately leading to unnecessary biopsy [6]. In addition, it also limited by its sensitivity, especially in women with dense breasts, as well as by other challenges and potential problems including radiation exposure and poor reproducibility [7–9].

Compared with mammography examination, ultrasound performs better in identifying dense breast. With the use of ultrasound, the detection sensitivity of small, invasive and lymph node negative breast cancer has been improved, especially in women with dense breast [10]. In China, women’s breasts are generally smaller and denser, ultrasound is an important examination method, which not only provides supplementary examination for abnormal mammography findings, but also an effective primary screening method for breast cancer [11]. Automated breast ultrasound (AB US) is an innovation in breast ultrasound. At present, some relevant systematic review articles have shown that AB US has the same or even higher diagnostic performance as handheld ultrasound (HHUS) [12–14]. Adding AB US as a screening tool for asymptomatic breast cancer in dense breast is more effective than performing mammography examination alone [10].

Advances in ultrasound imaging technology, especially the use of handheld high-frequency probes, have improved sonographic depiction of breast microcalcifications [15–17]. However, there are few reports on the evaluation of microcalcifications by AB US. To our knowledge, only a prospective study by Choi et al. reported that ABUS and HHUS have comparable detectability for suspicious calcification in a small sample (including 42 patients with 43 suspicious microcalcifications) [18]. The detection and diagnostic ability of AB US for breast microcalcification needs further exploration. Our team has conducted breast AB US examinations for over ten years, accumulating rich experience in diagnosing various breast diseases and storing a large amount of data. This study retrospectively included mammographically BI-RADS category 4 microcalcifications confirmed by pathology, analyzed and summarized AB US image features, and compared the evaluation results with mammography, aimed to explore the detection and diagnostic ability of AB US for these suspicious microcalcifications.

## Materials and methods

The present study was conducted following approval by the Institutional Review Board (IRB). Written informed consent was waived by the IRB due to the retrospective nature of the study.

### Study design and patient population

From January 2017 to June 2021, 2651 consecutive women with 2862 mamographically BI-RADS category 4 microcalcifications were collected.

**Fig. 1:**
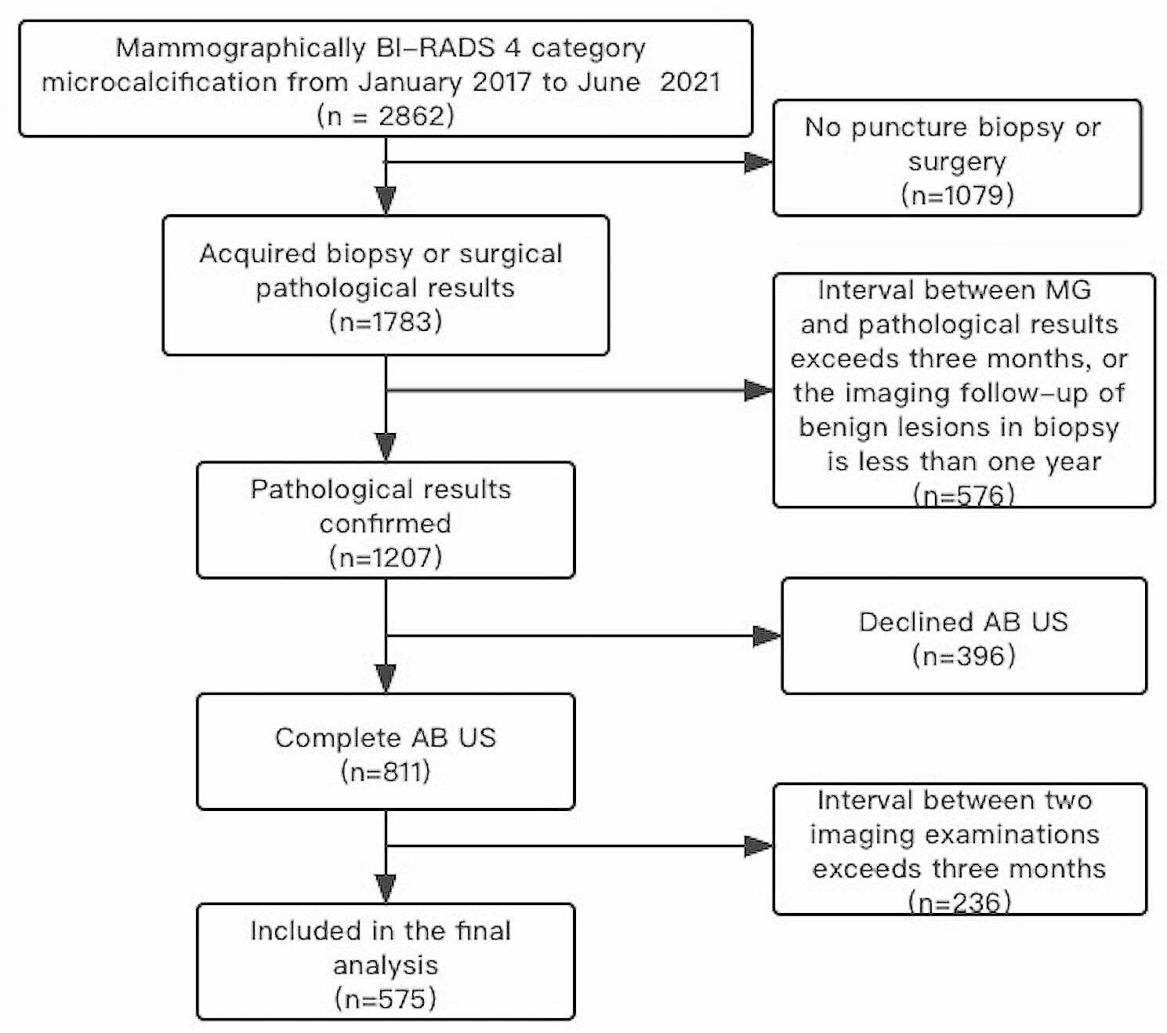
Flowchart shows enrollment of study participants. MG = mammography, AB US = Automated Breast Ultrasound

The inclusion criteria were: (1) pathologically confirmed by puncture biopsy or surgery; (2) the interval between mammography examination and pathological results were within three months, or the imaging follow-up of benign lesions in biopsy is more than one year; (3) completed AB US examinations; (4) the interval between mammography and AB US examinations were within three months. A total of 575 microcalcifications in 564 patients were included in the final analysis. Figure 1 described the flow of study enrollment.

### Image interpretation and reporting

Breast mammography was performed by an experienced technician with a standard devices (Hologic Selenia Dimensions, USA) to obtain standard craniocaudal and mediolateral oblique views. Image interpretation and reporting are independently completed by the experienced radiologists. Distinguished the breast density and focused on morphology and distribution of breast microcalcifications, the calcification morphology include punctate, amorphous, coarse heterogeneous, fine pleomorphic and fine-linear or branching, and calcification distribution include regional, grouped, linear and segmental. In addition, the presence of an associated mass, architectural distortion and asymmetries shadow were also analyzed. BI-RADS category was assigned according to the 5th Edition of BI-RADS Mammography, and BI-RADS category 4 was defined as a suspicious assessment [5].

AB US images were obtained using an ACUSON S2000 Automated Breast Volume Scanner (AB US, Siemens Medical Solutions, Mountain View, USA), which equipped with a 5–14 MHz wide aperture linear probe. The standardized scanning and image storing were completed by two experienced technicians, and image interpretation was independently completed by radiologists with 3 or more years of AB US diagnostic experience. Image interpretation and reporting was according to the 5th Edition of BI-RADS Ultrasound, calcification was divided into in a mass, outside of a mass and intraductal. Evaluation of calcification in a mass was based on the BI-RADS category of the mass, for mass with multiple microcalcifications, it should be appropriately upgraded. For microcalcifications outside of a mass or intraductal, they were often required to evaluate the associated features simultaneously, such as architectural distortion and duct changes, and ultimately assigned a BI-RADS category, BI-RADS category 4 and 5 were defined as positive.

### Reference standard

All lesions were confirmed by tissue biopsy or surgical pathology. Tissue biopsy includes ultrasound-guided coarse needle puncture biopsy and mammography guided stereotactic biopsy. If the biopsy indicated a malignant tumor and high-risk lesions (such as sclerosing adenopathy, atypical hyperplasia, mucocele, etc.), surgery was required, and the results were finally confirmed by surgical pathological results. While those confirmed as benign lesions after biopsy, relevant imaging follow-up was required for at least one year to maintain stability.

### Statistical analyses

SPSS 26.0 software (SPSS, Inc., Chicago, IL, USA) was used for recording data and statistical analyses. Measured data were presented as means ± standard deviations and categorical variables as percentages. Variables in different groups were compared by student’s t test or Chi-square test, and Bonferroni was further used for pairwise comparison of ratios among multiple groups. Univariate and multivariate logistic regression analysis were performed to explore the independent factors for predicting malignant microcalcifications and detecting mammographically BI-RADS category 4 microcalcifications by AB US. *P* value less than 0.05 was considered statistically significant.

## Results

### Patient characteristics

A total of 564 patients with 575 mammographically BI-RADS category 4 microcalcifications were included in this study. 250 (250/575, 43.5%) lesions were confirmed malignant, including 120 invasive breast carcinoma, 90 ductal carcinoma in situ, 25 ductal carcinoma in situ with invasive breast carcinoma, 7 solid papillary carcinoma, 6 mucinous carcinoma, 1 adenoid cystic carcinoma and 1 invasive lobular carcinoma, the remaining 325 (325/575, 56.5%) were benign, including 275 breast adenosis, 26 fibroadenoma, 5 intraductal papillomas, 2 inflammation, 1 fibroepithelioma, 2 mucocele, 3 atypical ductal hyperplasia and 11 sclerosing adenosis. All the patients were women, ranged in age from 24 to 86 years (average age, 48.40 ± 11.18 years). The average age of malignant group was (51.33 ± 11.93) year, older than (46.02 ± 9.91) year in benign group (*t* = 8.36, *P* < 0.001).

### Mammography findings between benign and malignant microcalcifications, and logistic regression analysis of malignant microcalcifications

Mammography findings between benign and malignant microcalcifications were analyzed. Microcalcifications morphology (61/80 with amorphous, coarse heterogeneous and fine pleomorphic, 13/14 with fine-linear or branching), distribution (189/346 with grouped, 40/67 with linear and segmental), associated features (53/77 with masses, 16/27 with architectural distortion, 70/96 with asymmetric shadow), higher BI-RADS category with 4B (88/120) and 4 C (73/38) showed higher incidence in malignant lesions (Table 1). In the logistic regression analysis, patients’ age (odds ratio [OR] = 1.05, *P* < 0.001), microcalcifications with amorphous, coarse heterogeneous and fine pleomorphic morphology (OR = 4.15, *P* < 0.001), fine-linear or branching morphology (OR = 13.71, *P* = 0.019), grouped distribution (OR = 5.01, *P* < 0.001), linear and segmental distribution (OR = 3.84, *P* = 0.001), associated with asymmetric shadow (OR = 2.49, *P* = 0.005), BI-RADS category 4B (OR = 5.73, *P* < 0.001) and 4 C (OR = 25.11, *P* < 0.001) were independently associated with malignant microcalcifications (Table 2).

**Table 1:** Mammography findings between benign and malignant microcalcifications

Mammography findings	Pathology results		*x2*, *P value*
	Benign (n = 325)	Malignant (n = 250)	
**Breast density**			*x*^*2*^ = 2.83, *P* = 0.092
a, b	15(2.6)	20(3.5)	
c, d	310(53.9)	230(40.0)	
**Calcification Morphology**			*x*^*2*^ = 58.14, *P* < 0.001
Punctate	305(53.0)	176(30.6)^a^	
Amorphous, Coarse heterogeneous, Fine pleomorphic	19(3.3)	61(10.6)^b^	
Fine-linear or branching	1(0.2)	13(2.3)^b^	
**Calcification Distribution**			*x*^*2*^ = 86.05, *P* < 0.001
Regional	141(24.5)	21(3.6)^a^	
Grouped	157(27.3)	189(32.9)^b^	
Linear, Segmental	27(4.7)	40(7.0)^b^	
**Associated findings**			*x*^*2*^ = 86.12, *P* < 0.001
No	264(45.9)	111(19.3)^a^	
Mass	24(4.2)	53(9.2)^b^	
Architectural distortion	11(1.9)	16 (32.8)^b^	
Asymmetric shadow	26(4.5)	70(12.2)^b^	
**BI-RADS category**			*x*^*2*^ = 183.80, *P* < 0.001
4A	288(50.1)	89(15.5)^a^	
4B	32(5.5)	88(15.3)^b^	
4C	5(0.9)	73(12.7)^c^	

Bonferroni was used for pairwise comparison in multiple groups. There were significant differences among the subsets (a, b, c)

**Table 2:** Uni-and multivariate logistic regression analysis of malignant microcalcifications

Variables	Univariate analysis		Multivariate analysis	
	OR (95%CI)	*P* value	OR (95%CI)	*P* value
**Age**	1.05 (1.03 ∼ 1.06)	< 0.001^*^	1.05 (1.03 ∼ 1.07)	< 0.001^*^
**Breast density**				
a, b	1.00 (Reference)		1.00 (Reference)	
c, d	0.56 (0.28 ∼ 1.11)	0.096	0.88 (0.36 ∼ 2.17)	0.780
**Calcification morphology**				
Punctate	1.00 (Reference)		1.00 (Reference)	
Amorphous, Coarse heterogeneous, Fine pleomorphic	5.56 (3.22 ∼ 9.62)	< 0.001^*^	4.15 (1.99 ∼ 8.65)	< 0.001^*^
Fine-linear or branching	22.53 (2.92 ∼ 173.67)	0.003^*^	13.71 (1.53 ∼ 122.95)	0.019^*^
**Calcification distribution**				
Regional	1.00 (Reference)		1.00 (Reference)	
Grouped	8.08 (4.88 ∼ 13.39)	< 0.001^*^	5.01 (2.71 ∼ 9.27)	< 0.001^*^
Linear, Segmental	9.95 (5.09 ∼ 19.43)	< 0.001^*^	3.84 (1.70 ∼ 8.69)	0.001^*^
**Associated features**				
No	1.00 (Reference)		1.00 (Reference)	
Mass	5.25 (3.09 ∼ 8.93)	< 0.001^*^	1.41 (0.69 ∼ 2.89)	0.350
Architectural distortion	3.46 (1.56 ∼ 7.69)	0.002^*^	0.52 (0.18 ∼ 1.51)	0.229
Asymmetric shadow	6.40 (3.88 ∼ 10.58)	< 0.001^*^	2.49 (1.32 ∼ 4.69)	0.005^*^
**BI-RADS category**				
4A	1.00 (Reference)		1.00 (Reference)	
4B	8.90 (5.57 ∼ 14.23)	< 0.001^*^	5.73 (3.31 ∼ 9.92)	< 0.001^*^
4C	47.24 (18.52 ∼ 120.55)	< 0.001^*^	25.11 (9.09 ∼ 69.40)	< 0.001^*^

OR: Odds Ratio; CI: Confidence Interval; * The differences were statistically significant

### Uni-and multivariate logistic regression analysis of the detectability of AB US for mammographically BI-RADS category 4 microcalcifications

477 (477/575, 83.0%) microcalcifications were detected by AB US, including 223 malignant and 254 benign, with a significantly higher detection rate for malignant lesions (*x*^2^ = 12.20, *P* < 0.001). Results of logistic regression analysis showed that microcalcifications with architectural distortion (OR = 0.30, *P* = 0.014), amorphous, coarse heterogeneous and fine pleomorphic morphology (OR = 3.15, *P* = 0.037), grouped (OR = 1.90, *P* = 0.017) and liner and segmental distribution (OR = 8.93, *P* = 0.004) were the independent factors which was associated with the detectability of AB US for these microcalcifications (Table 3).

**Table 3:** Uni-and multivariate logistic regression analysis of the detectability of AB US for mammographically BI-RADS category 4 microcalcifications

	Microcalcifications in AB US		x^2^/t, *P* value	Univariate analysis		Multivariate analysis	
	Detected (*n* = 477)	Not detected (*n* = 98)		OR (95%CI)	*P* value	OR (95%CI)	*P* value
**Age**	48.14 ± 11.48	49.23 ± 9.32	*t* = 4.92, *P* = 0.314	0.99 (0.97 ∼ 1.01)	0.378		
**Pathology results**			*x*^*2*^ = 12.20, *P* < 0.001				
Benign	254(44.2)	71(12.3)		1.00 (Reference)		1.00 (Reference)	
Malignant	223(38.8)	27(4.7)		2.31 (1.43 ∼ 3.72)	< 0.001^*^	1.68 (0.95 ∼ 2.96)	0.075
**Breast density**			*x*^*2*^ = 5.46, *P* = 0.020				
a, b	24(4.2)	11(1.9)		1.00 (Reference)		1.00 (Reference)	
c, d	453(78.8)	87(15.1)		2.39 (1.13 ∼ 5.05)	0.023^*^	3.06 (1.32 ∼ 7.08)	0.009^*^
**BI-RADS category**			*x*^*2*^ = 8.76, *P* = 0.013				
4A	302(52.5)	75(13.1)^a^		1.00 (Reference)			
4B	102(17.7)	18(3.1)^a, b^		1.41 (0.80 ∼ 2.47)	0.233		
4C	73(12.7)	5(0.9)^b^		3.63 (1.42 ∼ 9.29)	0.007*		
**Associated features**			*x*^*2*^ = 5.74, *P* = 0.125				
No	307(53.4)	68(11.8)		1.00 (Reference)		1.00 (Reference)	
Mass	68(11.8)	9(1.6)		1.67 (0.80 ∼ 3.52)	0.174	1.47 (0.62 ∼ 3.47)	0.383
Architectural distortion	19(3.3)	8(1.4)		0.53 (0.22 ∼ 1.25)	0.146	0.30 (0.11 ∼ 0.78)	0.014^*^
Asymmetric shadow	83(14.4)	13(2.3)		1.41 (0.74 ∼ 2.68)	0.289	0.89 (0.44 ∼ 1.81)	0.746
**Calcification Morphology**			*x*^*2*^ = 13.71, *P* = 0.001				
Punctate	388(67.5)	93(16.2)^a^		1.00 (Reference)		1.00 (Reference)	
Amorphous, Coarse heterogeneous, Fine pleomorphic	76(13.2)	4(0.6)^b^		4.55 (1.62 ∼ 12.76)	0.004^*^	3.15 (1.07 ∼ 9.23)	0.037^*^
Fine-Linear or branching	13(2.3)	1(0.2)^a, b^		3.12 (0.40 ∼ 24.12)	0.276	1.17 (0.14 ∼ 9.75)	0.885
**Calcification Distribution**			*x*^*2*^ = 22.09, *P* < 0.001				
Regional	118(20.5)	44(7.7)^a^		1.00 (Reference)		1.00 (Reference)	
Grouped	194(33.7)	52(9.0)^b^		2.11 (1.34 ∼ 3.32)	0.001^*^	1.90 (1.12 ∼ 3.22)	0.017^*^
Linear, Segmental	65(41.3)	2(0.3)^c^		12.12 (2.85 ∼ 51.61)	< 0.001^*^	8.93 (2.04 ∼ 38.96)	0.004^*^

OR: Odds Ratio; CI: Confidence Interval; * The differences were statistically significant; Bonferroni was used for pairwise comparison in multiple groups. There were significant differences among the subsets (a, b, c)

### AB US fingdings in 477 detected microcalcifications

Among the microcalcifications detected by AB US, malignant lesions were more common in a mass and intraductal. And malignant microcalcifications tend to be associated with duct change or architectural distortion or both of all. There were 12 microcalcifications associated with both architectural distortion and duct changes, and all were ultimately confirmed to be malignant. AB US findings between malignant and benign microcalcifications were summarized in Table 4.

**Table 4:** AB US fingdings in 477 detected microcalcifications lesions

AB US findings	Pathology results		x^2^, *P* value
	Benign (*n* = 254)	Malignant (*n* = 223)	
**Calcification Distribution**			*x*^*2*^ = 48.85, *P* < 0.001
Outside of a mass	192(40.3)	99(20.7)^a^	
Inside a mass	50(10.5)	104(21.8)^b^	
intraductral	12 (2.5)	20(4.2)^b^	
**Associated findings**			*x*^*2*^ = 87.03, *P* < 0.001
No	233(48.8)	123(25.7)^a^	
Architectural distortion	10(2.1)	58(12.1)^b^	
Duct changes	11(2.3)	30(6.3)^b^	
Architectural distortion and duct changes	0(0.0)	12(2.5)^b^	
**BI-RADS category**			*x*^*2*^ = 117.53, *P* < 0.001
0	67(14.0)	7(1.5)^a^	
2 and 3	54(11.3)	1(0.2)^a^	
4 and 5	133(27.9)	215(45.1)^b^	

Bonferroni was used for pairwise comparison in multiple groups. There were significant differences between the subsets (a, b)

### AB US and mammography BI-RADS categories of 477 detected microcalcifications

BI-RADS categories between AB US and mammography were partial different in the 477 detected microcalcifications (Table 5). 74 were classified as category 0, 55 were downgraded to category 2 or 3, and 23 were upgraded to category 5, the remaining 325 were maintain diagnosis of category 4 by AB US. The sensitivity of AB US to diagnose malignant lesions was 64.8% (226/349), higher than 46.8% (223/477) of mammography.

**Table 5:** AB US and mammography BI-RADS categories of 477 detected microcalcifications

Pathology results	AB US BI-RADS category						Mammography BI-RADS category		
	0	2 and 3	4 A	4B	4 C	5	4 A	4B	4 C
benign	67	54	123	10	0	0	224	26	4
malignant	7	1	27	64	101	23	78	76	69
Total	74	55	150	74	101	23	302	102	73

## Discussion

Microalcification has high specificity for breast cancers, up to 86% could be detected by mammography, and it has been widely used for the detection and diagnosis of breast calcifications [19–21]. The 5th edition of BI-RADS Mammography has formulated established diagnostic criteria and recommended protocols based on the morphology and distribution characteristics of calcifications, which are important factors in identifying benign and malignant lesions. Amorphous, coarse heterogeneous and fine pleomorphic morphology calcifications are suggested a moderate suspicion of malignancy. In our study, quantitative risk was assigned with OR = 5.56 in univariate analysis and 4.15 in multivariate analysis. Fine-linear or branching calcifications are considered high suspicion of malignancy, and OR = 22.53 in univariate analysis and 13.71 in multivariate analysis was assigned. Distribution with grouped, linear and segmental distribution are also confirmed to associated with malignant. In addition, mass, architectural distortion and asymmetric shadows are also important mammography features, compared with pure microcalcifications, the presence of these findings significantly increased the risk of malignancy [22], which was confirmed in our univariate analysis, and asymmetric shadows was the independent risk for malignant lesion.

In the study, AB US was analyzed, for its new ultrasonic examination mode (technicians standardize scanning and image storing, and sonographer reading), and overcame the shortcomings of HHUS, such as relative dependence on operator experience, low repeatability, small imaging area, and lack of coronal images. It also allowed full ultrasound research in screening dense and extremely dense breast cancer [23]. Asian women have relatively dense breast, and 93.9% (540/575) of the patients have dense and extremely dense breasts in this study. 83.0% (477/575) were detected by AB US, which was higher than 74.4% in Choi et al.’s study. Similarly, the detection rate of malignant calcification was significantly higher than that in benign lesions (88.7% vs. 78.1%) [18], which were displayed with architectural distortion, amorphous, coarse heterogeneous and fine pleomorphic morphology, and distributed with grouped, linear and segmental.

According to the 5th edition of BI-RADS Ultrasound, breast calcification has been listed in a separate catalogue and classified as in a mass, outside of a mass and intraductal [16]. Malignant microcalcification in a mass and intraductal were more commonly detected By AB US in our study. It is likely that the reduction of echo background increased its detectability, while microcalcification outside of a mass are often located in adipose or fibrous glandular tissue, without shadows, making it less likely to be detected.

**Fig. 2:**
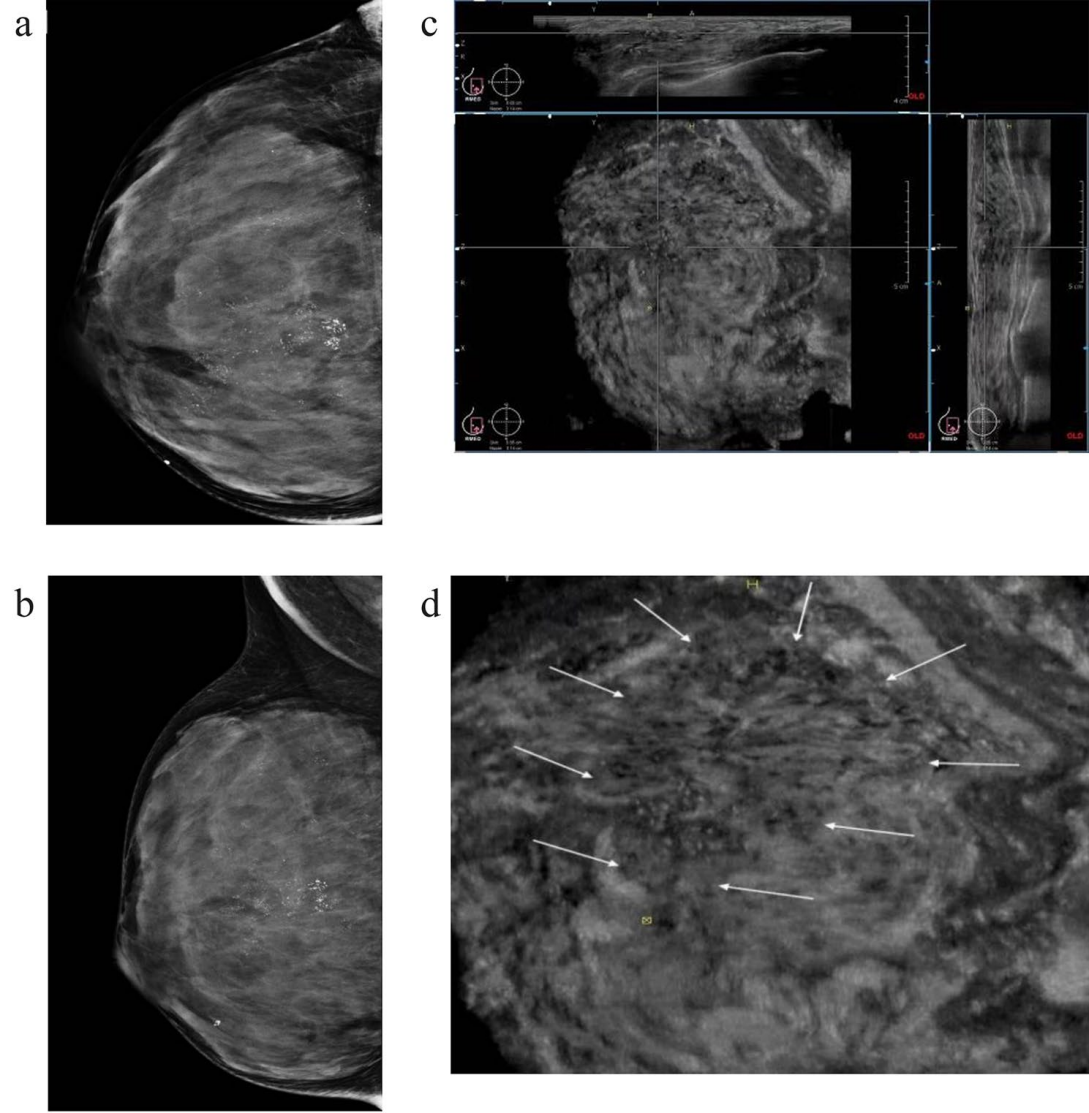
Images of a young woman with microcalcifications in right breast, which pathologically confirmed ductal carcinoma in situ with invasive breast carcinoma. **a, b** Mammography CC and MLO views show multiply fine pleomorphic and branching calcifications segmental distributed in the upper and central district of right breast. Breast density is d and assessed as BI-RADS category 4 C. **c** Right AB US MED plane shows multiply dilated catheters with microcalcifications intraductal on the coronal (lower left), transverse (top) and sagittal plane images (right). Assessed as BI-RADS category 4 C. Nipple is marked as yellow dot, the crosshairs locate the lesions. **d** shows the lesions segmental distributed on the right MED coronal plane (arrows)

**Fig. 3:**
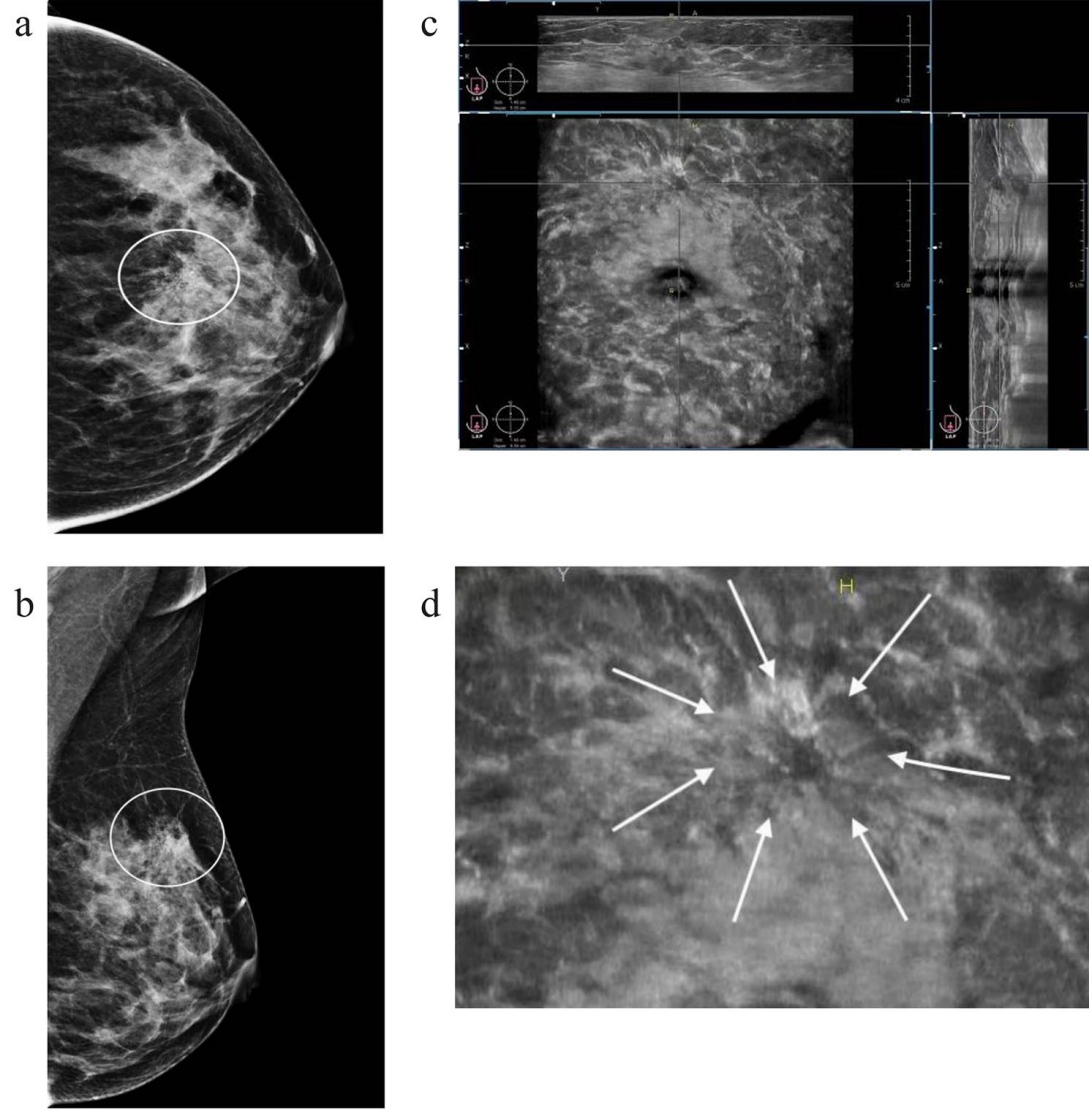
Images of a middle-aged woman with microcalcifications in left breast, which pathologically confirmed ductal carcinoma in situ with microinvasion. **a, b** Mammography CC and MLO views show multiply punctate calcifications grouped distributed in the upper quadrant of left breast with architectural distortion. Breast density is c and assessed as BI-RADS category 4 C. **c** Left AB US AP plane shows multiply microcalcifications outside of a mass with architectural distortion on the coronal (lower left), transverse (top) and sagittal plane images (right). Assessed as BI-RADS category 4 C. Nipple is marked as yellow dot, the crosshairs locate the lesions. **d** shows the retraction phenomenon on the AP coronal plane (arrows)

AB US contains the whole breast volume data, and can be continuously observed in multiplanar observation, the large field of view and unique coronal planes not only fully display the overall distribution and interrelationships of single or multiple lesions, but also help to visualize the discovery of ductal related lesions and architectural abnormalities [24, 25]. Calcification presents a linear or segmented distribution on mammography, often indicating deposition of calcification in one or more ducts and branches. The AB US coronal plane provides a new perspective for intraductal lesions, 65 of the 67 microcalcifications with linear or segmental distribution were detected by AB US (Fig. 2). Retraction phenomenon is an important sign of architectural distortions on the coronal view of AB US, characterized by a radial distribution of high echogenicity around the lesion converging towards the center, with high sensitivity and specificity for cancer [25–27] (Fig. 3). The sign can only be displayed at partial layer, and invasive breast cancer with small focus, shallow and low histological grade is more likely to occur. In this study, 68 cases showed a retraction phenomenon, 58 were confirmed as malignant, and the other 10 cases were benign lesions, including 8 adenopathy and 2 atypical hyperplasia, which can also cause damage to surrounding normal tissues [28]. Notably, 12 lesions exhibited both with ductal changes and retraction phenomenon, which ultimately confirmed as malignant lesions. Therefore, the coexist of retraction phenomenon and ductal changes in calcified lesions can greatly improve the diagnostic confidence for malignant lesions.

The BI-RADS categories of AB US and mammography for each lesion were compared. In ultrasound images, bilateral and scattered calcifications outside the duct as well as calcifications related to fibrocystic lesions, are usually considered benign and classified as BI-RADS category 0 or 2. For calcifications that accumulate outside the duct, BI-RADS class 0 is recommended, and further imaging examinations were recommended. In addition, evaluation of the calcification in a mass was based on the BI-RADS category of the mass, and after comprehensive consideration, it can also be upgraded or downgraded accordingly. Therefore, out of the 477 lesions detected by AB US for calcification, 74 were classified as category 0, 55 were downgraded to category 2 or 3, 23 were upgraded to category 5, and the remaining 325 were maintain category 4. Compared with mammography, AB US has a higher sensitivity (64.8% vs. 46.8%) for malignant calcification.

There are some shortcomings in this study. Firstly, only pathologically confirmed mammographically BI-RADS category 4 microcalcifications in a single center were included, the patient selection might biased. Secondly, this study focused on dense and extremely dense breasts, and the research results might not suitable for all populations. Thirdly, this is a retrospective observation study, all image interpretation and reporting results of the breast mammography and AB US derived from existing records, and the order of the two examinations was random and the results were not all double-blind. Among them, 365 mammography examination results were complete earlier than AB US, and 210 were later than AB US. The detection rate of AB US for calcification was significantly higher (326/365 vs. 151/210) with knowledge of the mammography results, but no significant difference in detection of malignant lesions (165/365 vs. 88/210).

## Conclusions

AB US has good detectability for BI-RADS category 4 suspicious microcalcificationa, especially for malignant calcification lesions. Malignant calcification is more common in a mass and intreductral in AB US, and when associated with architectural distortion or duct changes, it is beneficial for the diagnosis of malignant lesions. Compared to mammography examination, ABUS has higher sensitivity to malignant calcification, especially in dense breast, which is expected to become an effective supplementary examination method.

## Data Availability

The datasets used and analyzed during the current study are available from the corresponding author on reasonable request.

## Abbreviations

AB US: Automated Breast Ultrasound
ACR: American College of Radiology
BI-RADS: Breast Imaging Reporting and Data System
HHUS: Handheld ultrasound
IRB: Institutional Review Board
OR: Odds Ratio
